# Cucumarioside A_2_-2 Causes Macrophage Activation in Mouse Spleen

**DOI:** 10.3390/md15110341

**Published:** 2017-11-01

**Authors:** Evgeny A. Pislyagin, Igor V. Manzhulo, Tatiana Y. Gorpenchenko, Pavel S. Dmitrenok, Sergey A. Avilov, Alexandra S. Silchenko, Yun-Ming Wang, Dmitry L. Aminin

**Affiliations:** 1 G.B. Elyakov Pacific Institute of Bioorganic Chemistry, Far Eastern Branch of the Russian Academy of Sciences, 690022 Vladivostok, Russia; paveldmt@piboc.dvo.ru (P.S.D.); avilov-1957@mail.ru (S.A.A.); sialexandra@mail.ru (A.S.S.); daminin@piboc.dvo.ru (D.L.A.); 2 National Scientific Center of Marine Biology, Far Eastern Branch of the Russian Academy of Science, 690041 Vladivostok, Russia; manzhulo.iv@dvfu.ru; 3 School of Biomedicine, Far Eastern Federal University, 690091 Vladivostok, Russia; 4 Federal Scientific Center of the East Asia Terrestrial Biodiversity, Far Eastern Branch of the Russian Academy of Sciences, 690022 Vladivostok, Russia; gorpenchenko@biosoil.ru; 5 Department of Biological Science and Technology, Institute of Molecular Medicine and Bioengineering, National Chiao Tung University, Hsinchu City 300, Taiwan; ymwang@mail.nctu.edu.tw

**Keywords:** immunomodulation, LPS, iba-1, IL-1β, NO, iNOs, ROS, sea cucumbers, spleen, triterpene glycosides

## Abstract

The immunomodulatory effect of triterpene glycoside cucumarioside A_2_-2 (CA_2_-2), isolated from the Far Eastern sea cucumber *Cucumaria japonica*, was compared with lipopolysaccharide (LPS) on mouse spleen. It has been shown that the intraperitoneal (*i.p.*) glycoside administration leads to increased spleen macrophage activating markers iba-1, IL-1β, iNOs, ROS and NO formation, with additional change of macrophage phenotype to M1. The mass spectrometry profiles of peptide/protein were obtained using MALDI-TOF-MS on the different parts of spleen sections isolated by laser mircodissection techniques. It was found that *i.p.* stimulation of animals with CA_2_-2 leads to marked changes in the intensity of the characteristic peaks of spleen peptides/proteins, primarily in red pulp.

## 1. Introduction

The triterpene glycoside cucumarioside A_2_-2 (CA_2_-2) isolated from the Far-Eastern edible sea cucumber *Cucumaria japonica* has been found to have pronounced immunomodulatory effects in vivo [1,2,3]. Using inhibitory analysis, patch-clamp on single macrophages, small interfering RNA technique, immunoblotting, SPR analysis, computer modeling and other methods, we recently demonstrated that low doses of CA_2_-2 specifically interact with P2X receptors on membranes of mature macrophages, enhancing the reversible ATP-dependent Ca^2+^ intake and recovery of Ca^2+^ transport inactivation of these receptors. As a result, interaction of glycosides of this type with P2X receptors leads to activation of cellular immunity [4].

It is known that one of the target organs for CA_2_-2 immunomodulatory action is the spleen. Previously, by MALDI-TOF-MS, MALDI-IMS and radiospectroscopic studies of ^3^H-CA_2_-2, we determined the quantitative content and pharmacokinetics of CA_2_-2 in mouse spleen after drug *i.p.* administration [5]. Cucumarioside A_2_-2 was absorbed fairly rapidly; the glycoside maximum concentration (C_max_) in spleen was observed in the first 30 min after injection, and the biological half-life (T½) of the compound was approximately 80 min. The mean residence time of the preparation (MRT) was calculated to be approximately 140 min. It was established that CA_2_-2 localized in the tissue at high concentrations in the regions surrounding the organ followed by its decline on the surface and by a very slight redistribution to the internal portions of the spleen.

The in vivo study of CA_2_-2 immunomodulatory action was carried out in comparison with LPS. The glycoside *i.p.* administration resulted in a tendency towards an increase in splenic weights compared with those from control mice receiving PBS alone. Changes in the ratio of red to white pulp after CA_2_-2 or LPS administration were observed. The proportion of splenic white pulp after glycoside or LPS administration increased by up to 34% and 36%, respectively. A detailed study of the distribution of the PCNA marker showed that the proliferative activity in the white pulp under CA_2_-2 and LPS influence increased 2.07 and 2.24 times, respectively. The mass spectrometry profiles of spleen peptide/protein homogenate were obtained using the MALDI-TOF-MS approach. It was found that *i.p.* stimulation of animals with CA_2_-2 or LPS leads to marked changes in the intensity of the protein/peptide peaks generally accepted as representative of exposure to immunostimulants in general [6].

However, despite a large number of studies of the physiological activity of holothurian triterpene glycosides, the immunomodulatory mechanism of their action on the organ and clinically relevant biomarkers for immunostimulation has not been sufficiently studied. The aim of this study is to continue the detailed investigation of the immunomodulatory effect of CA_2_-2 in in vivo experiments. For this purpose, the effect of CA_2_-2 and LPS upon iba-1, IL-1β, ROS and NO production, and iNOs expression in macrophage spleen was studied by immunohystochemistry and spectrofluorimetric approaches. Using laser microdissection and MALDI-TOF-MS techniques, the MS spectra of red and white pulp of the spleen were obtained and clinically relevant immunomodulatory biomarkers were compared.

## 2. Results

### 2.1. CA_2_-2 Increased iba-1, IL-1β and iNOs Positive Stained Areas in Red Pulp of Mouse Spleen

Ionized calcium-binding adapter molecule 1 (iba-1) or allograft inflammatory factor 1 (AIF-1) is a transient protein that is highly expressed in the testicles and spleen, but is weakly expressed in the brain, lungs and kidneys [7]. It was shown that the iba-1 gene is selectively expressed in microglia and macrophages of activated tissue [8]. Macrophages are one of the key links in immunity; they act as the first line of defense in the immune system against pathogens, and play a major role in maintaining tissue homeostasis. One of the biomarkers by which activated macrophages can be determined is iba-1.

In the spleen sections of the control group of animals, after coloring with antibodies, a brown background (red pulp of the spleen) is seen in which the contours of lymphoid nodules are clearly visible. They have a rounded shape, but a different size. Inside, central arterioles located eccentrically are seen near which iba-1 positive cellular structures are also observed.

**Figure 1:**
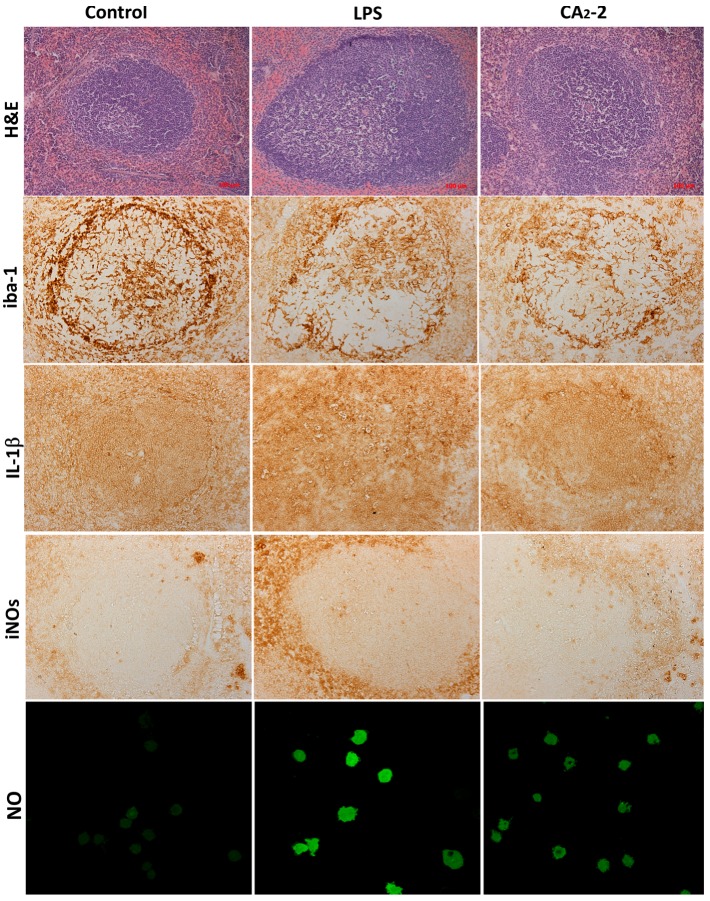
Sections of Balb/c mouse spleens, stained with H&E, iba-1, iNOs, IL-1β, and NO determination in spleen macrophages. Control, CA_2_-2 treatment and LPS treatment are shown.

Iba-1positive cellular structures are homogeneous, intensely brown in color, star-shaped, with multiple thin branching outgrowths. The maximum concentration of iba-1 positive cells was found in the marginal zone and near PALS, and in the germinal center. The structural organization of most identified iba-1-positive cells of the white and red pulp of the spleen of mice makes it possible to identify these cells as macrophages. They have a characteristic localization and shape, and a peculiar branching of the outgrowths (Figure 1).

**Figure 2:**
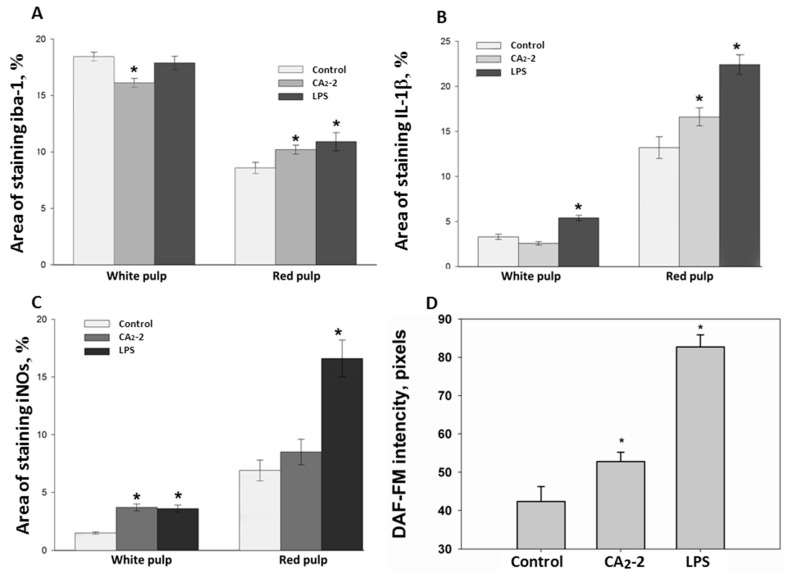
Immunohistochemical quantitative determination of iba-1 (**A**); IL-1β (**B**); iNOs (**C**) positive cells in Balb/c spleen sections after CA_2_-2 or LPS treatment in the white and red pulp; NO content determination in spleen macrophage primary culture (**D**). Data are presented as m ± se, * *p* < 0.05, *n* = 60 (one-way ANOVA).

As shown on Figure 2A, the area of stained iba-1 positive cells in white pulp is larger than the area of staining in the red pulp. This ratio is observed in all studied groups (control, glycoside treated, LPS treated). However, the mice receiving glycoside have a staining area in white pulp significantly less by 12.7% of the control area. At the same time, an increase in the iba-1 staining area is observed in red pulp of spleen of animals treated with CA_2_-2 and LPS by 18.6% and 27.1%, respectively, compared to control red pulp area. Visually, it can be noted that the marginal zone and PALS in the sections of the spleens of glycoside- and LPS-treated animals seem less contoured than in the control.

Immunohistochemical staining of the spleen sections with antibodies to IL-1β (Figure 2B) showed that the area of staining in the red pulp is more than 3 times greater than in the white pulp. The area of staining of the white pulp did not significantly change when mice were injected with glycoside, but increased after LPS administration up to 63.6% of the control area. In the red pulp, the IL-1β staining area is significantly higher in spleens of animals receiving the glycoside or LPS by 25.7% and 69.7% of the control, correspondingly.

Histochemical staining of the spleen sections with antibodies to iNOs and analysis of the sections (Figure 2C) indicated that the staining area in the red pulp is more than 3 times greater than in the white pulp (control, glycoside and LPS group). The increase in the iNOs positive area in white pulp of mice treated with glycoside and LPS by 146% and 140% was observed. In the red pulp, the area of antibody staining to iNOs is significantly higher. In animals treated with LPS only, the iNOs positive area was increased by 196.7% of the control. In CA_2_-2 treated animals, only a non-significant tendency to increase by 23% was found in the red pulp.

### 2.2. CA_2_-2 Stimulates NO and ROS Production in Spleen Macrophages

We investigated the influence of immunomodulators on the nitric oxide production in the mouse spleen macrophages. Fluorescent staining of spleen macrophages with a fluorescent probe DAF-FM sensitive to NO (Figure 1 and Figure 2D) showed that the cellular fluorescence intensity increases in the glycoside treated mice (by 36%) as well as in the LPS treated mice (by 111%) after compound *i.p.* administration.

**Figure 3:**
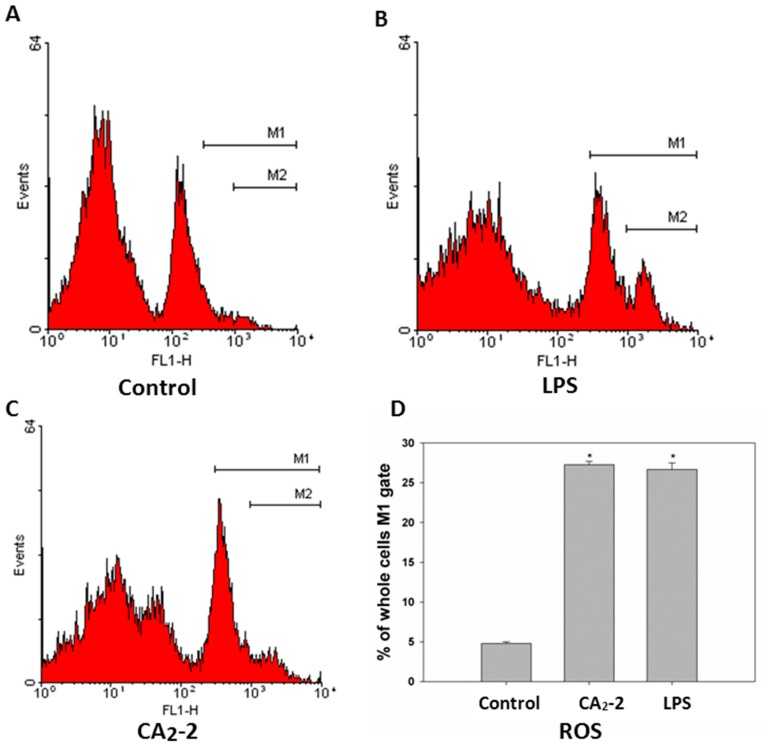
Flow cytometry analysis of ROS generation in spleen macrophages of Balb/c mice treated with LPS and CA_2_-2 for 18 h. The DCF fluorescence histograms (**A**–**C**) and ROS content in cells (**D**) are shown. * *p* < 0.05.

To measure the ROS formation in mouse spleen macrophages we used a DCFDA fluorescent dye coupled with flow cytometry. It was found that spleen macrophages consist of at least two subpopulations of cells differing in size and granulation. It can be noted that the cells also differ in fluorescence intensity. There is the cell subpopulation with low ROS content and a smaller portion of cells with relatively higher level of ROS formation (Figure 3). It was shown that the treatment of mice with CA_2_-2 of LPS leads to an increase in macrophage number with relatively higher ROS content. Both immunostimulants exhibit approximately a 5-fold increase in the ROS formation in macrophages compared to control animal group.

### 2.3. CA_2_-2 Induce Macrophage Activation and M1 Polarization

**Figure 4:**
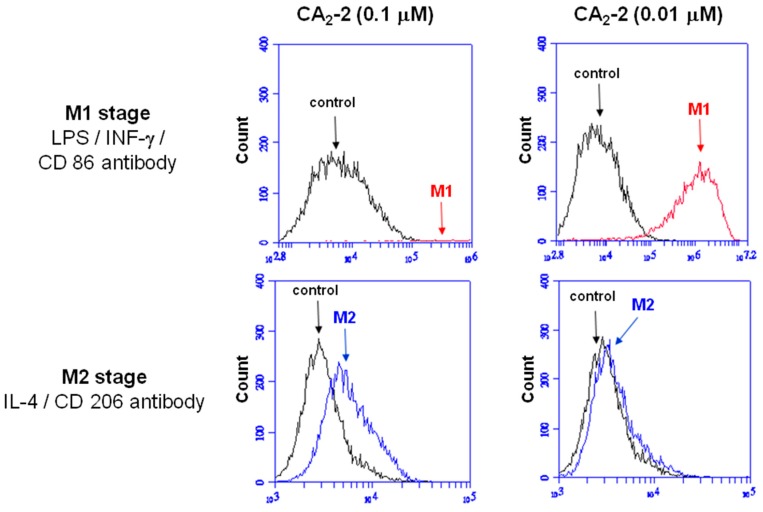
Macrophage M1/M2 polarization and activation. Flow cytometry analyses of RAW 264.7 cells treated with CA_2_-2 (0.1 or 0.01 μM), following by LPS (1 μg/mL) and IFN-γ (20 ng/mL) for M1 polarization or IL-4 (20 ng/mL) for M2 polarization.

As shown in Figure 4, the fluorescence signal was obviously shifted (from 10^4^ to 10^6^) when RAW 264.7 macrophages co-incubated with 0.01 μM CA_2_-2, while the number of shifted cells were very few by 0.1 μM A_2_-2 treatment. These results demonstrated that low concentration of CA_2_-2 was more efficient in M1 polarization. At the same time, the M2 polarization stage of macrophages was not significantly induced by CA_2_-2 application.

### 2.4. CA_2_-2 Induces a Shift in Protein/Peptide Profile of Spleen Tissue

To study the localization of potential target proteins in the spleen, a laser microdissection method was used. This method allows isolating and obtaining individual cells, groups of cells or selected tissue sites from the tissue slices.

**Figure 5:**
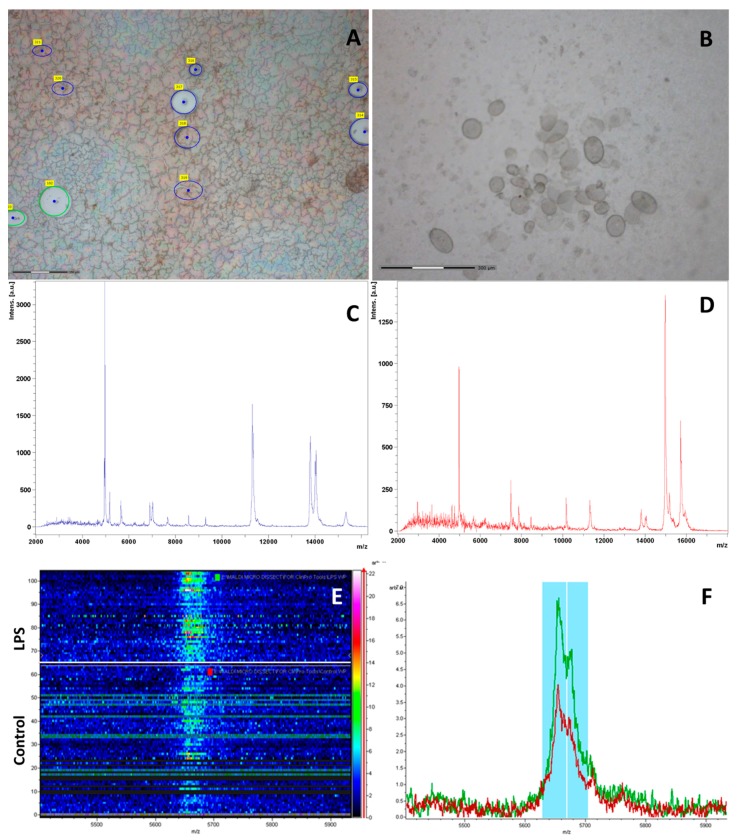
The process of cutting red and white pulp in the mouse spleen section by laser microdissection (Carl Zeiss PALM MicroBeam). Green circles indicate the white pulp excision area; blue circles indicate the red pulp excision area. It can be seen that some of the selected tissue sections have already been cut (**A**); The bottom of the tube cap with cut out and collected tissue sections (**B**); Representative MALDI-TOF mass spectra of the mouse spleen samples from white pulp (**C**) and red pulp (**D**); The protein profiles of mouse treated with CA_2_-2 are shown. The pseudo-gel view of representative mass spectra of two different mouse spleen samples (Control and LPS) with all individual spectra shown in a density scale, in the mass range from 5000 to 6000 Da (**E**) and corresponding mass spectra profile, showing significant differences between untreated and LPS treated animal samples (**F**).

Red and white pulp samples of the spleen are of particular interest, since it is in these areas that the main morphological and biochemical changes occur when the immune system is activated. It is well known that the majority of macrophages are located in the marginal zone and red pulp of the spleen. Accordingly, the sections of the red and white pulp (including marginal zone, MZ) were cut from the sections of the spleens isolated from the mice of control group, and CA_2_-2 and LPS treated groups. All samples from the spleen were cut with the same total area (600 μm^2^) to obtain the same amount of protein in the samples (Figure 5A,B).

An identification and analysis of mass peaks in the red and white pulps was conducted using MALDI-TOF-MS on these tissue samples obtained 18 h after drug administration. A profile of proteins and peptides detected in the red and white pulp tissue of the CA_2_-2 treated group is presented on Figure 5C,D as an example.

The ClinProTools software was used for the data interpretation of MALDI-TOF spectra derived from spleen samples of different experimental groups. A representation of the signal intensities in a rainbow color scale allowed for detection of faint differences between the classes due to the changing colors with increasing peak height (Figure 5E,F). Statistical analysis of MALDI-TOF mass spectra of spleen samples at different stimulants allowed the identification of discriminant peaks based on their signal intensity. A class prediction model was set up by GA; marker polypeptides/proteins were identified by means of their PWKW *p*-values.

**Control vs. CA_2_-2:** The results of analysis of the average mass spectra and the statistical processing of the white pulp spleen samples of CA_2_-2 and control groups of animals, presented in Table 1, showed that 3 peaks with *m*/*z* 4618.93, 6109.73 and 14,969.48 had significantly different signal levels (*p* < 0.05). It was noted that the expression of two these peptides in the white pulp was decreased while there was also a peptide whose intensity was increased.

In the red pulp, 13 peaks had significantly different signal levels in spleen samples of CA_2_-2 and the control group. The expression of all peptides was increased, including those peaks that were significantly decreased in the white pulp. The most important regulated peptides corresponded to the peaks with *m*/*z* 10,171.16, 15,722.66, 15,938.71, 15,181.67, 16,025.7, 15,347.37, 15,298.01 and 15,321.28. Two peaks of them with *m*/*z* 10,171.16 and 15,722.66 had the highest *p* values.

**Control vs. LPS:** In the white pulp, 12 peaks had significantly different signal levels (*p* < 0.05) in the mass spectra of the LPS and control spleen samples (Table 2). It was noted that, after LPS administration was increased, the expression of all peptides in the white pulp was increased, except the peaks with *m*/*z* 7483.7. The most notable peaks regulated in by LPS in white pulp were *m*/*z* 5673.7, 11,315.51, 7026.31, 7483.79, 11,353.64, 14,055.76 and 5655.6.

In the red pulp, 14 peaks were found to regulate by LPS with a very high significance value. Surprisingly, the expression of all regulated peptides in the red pulp of the spleen were increased, except the peak with *m*/*z* 4966.

## 3. Discussion

In present study, sections of the mouse spleen were stained with antibodies to iba-1. This peptide was found in the cells of the marginal zone of the lymph nodes, red pulp, as well as in the germinal center and in the periarteriolar lymphoid sheaths (PALS) in control animals, and after 18 h of the CA_2_-2 or LPS administration. It has previously been found that the antibody staining area for iba-1 increases after the administration of immunostimulants, such as LPS [9]. This agrees well with our results. Similarly to LPS application, CA_2_-2 treatment leads to an increase in the area of cells positively stained with antibodies to iba-1 in the red pulp of the spleen. At the same time, in the spleen of mice receiving CA_2_-2, a significant decrease in this marker in white pulp was observed. It can be assumed that such redistribution occurs as a result of the intensification of activated macrophage migration in the spleen from the zone of white pulp to the red pulp. Recently, it was shown that CA_2_-2 is able to effectively stimulate the adhesion, spreading and motility of macrophages [10].

Previously, it was shown that a certain level of endogenous iba-1 is expressed in macrophages and its level is increased by the stimulation of these cells with the IL-1β or TNF-α cytokines [11]. IL-1β is expressed in spleen or peritoneal macrophages by stimulation with LPS [12,13] and by microbial infection [14,15]. In the current investigation, we have shown that the glycoside causes an increase in the IL-1β staining area mainly in the red pulp, which may indicate activation of macrophages localized predominantly in the red pulp of the spleen.

Bacterial LPS is known to cause endotoxic shock, iNOs expression and the formation of nitric oxide (NO). NO plays an important role in mammalian immunosurveilance and is produced by macrophages during bacterial resistance. It has been shown that the lungs, spleen and kidneys are the main focii of iNOs expression during endotoxic shock [16]. We have shown that LPS causes a significant increase in iNOs, both in the red and white pulp of the spleen, which indicates the full launch of the inflammatory response. In the case of CA_2_-2, a significant increase in iNOs is observed in white pulp, while in red pulp only a tendency to increase NO synthase expression is observed.

The ability of macrophages of mice activated with lipopolysaccharide to synthesize NO_2_ and NO_3_ ions was detected in 1985 [17]. Later it was shown that NO metabolites are formed as a result of the induction of Ca-independent NO synthase, which in humans and animals is the most effective source of NO. The activity of this enzyme, which was later called inducible NO synthase, is 100 times greater than that of the endothelial enzyme. The formation of NO by macrophages is stimulated by bacterial lipopolysaccharides, as well as lymphokines of T-lymphocytes, such as interleukin-1 and interleukin-2, interferon-γ or its combination with TNF-α, TNF-β and lipid A [18,19]. It was found that CA_2_-2 and LPS cause an increase in NO production in spleen macrophages. Moreover, it was noted that the formation of nitric oxide in macrophages by this glycoside is noticeably less than in cases with LPS. This is consistent with published data on the effect of these immunostimulants on iNOs expression. Taken together, this is indicative of the moderation of this glycoside’s immunostimulatory effect.

Phagocytes (neutrophils and macrophages) use reactive oxygen species (ROS) for the destruction of microorganisms. Macrophages destroy damaged, old or immunologically incompatible cells, and also contribute to the destruction of malignant cells and cells infected by viruses, as well as bacterial cells. We observed a significant and approximately equal increase in the ROS formation in spleen macrophages of animals receiving LPS or CA_2_-2. This indicates an activation of macrophages and pronounced immunostimulatory effect by these agents. In accordance with the «M1/M2» paradigm, two subtypes of activated macrophages are distinguished-classically activated (M1) and alternatively activated (M2), which express different receptors, cytokines, chemokines, growth factors and effector molecules. Classically activated macrophages (M1 phenotype) are stimulated by IFN-γ, as well as IFN-γ in conjunction with LPS and TNF. Their main functions are the destruction of pathogenic microorganisms and the induction of an inflammatory reaction. Along with pro-inflammatory cytokines such as cytokine IL-1β, macrophages secrete anti-inflammatory cytokine-IL-10, with a characteristic high IL-12/IL-10 ratio. Alternative activation of macrophages (M2 phenotype) is observed when stimulated with interleukins, glucocorticoids, immune complexes, TLR agonists, etc. For macrophages of this type, a low IL-12/IL-10 ratio is observed [20]. In our experiments it was shown that CA_2_-2 induces activation and polarization in macrophages precisely according to the classical M1 type (not M2 type). This is in good agreement with the results showing an increase in the production of ROS, NO, IL-1β, activation of iNOs and iba-1 in spleen macrophages by this glycoside, corresponding to their activation by the classical pathway.

The spleen is the largest secondary immune organ in mammals and is responsible for initiating an immune response to blood-borne antigens and for filtering the blood of foreign material and old or damaged red blood cells. These functions are carried out by the two main compartments of the spleen, the white pulp (including the marginal zone) and the red pulp, which are vastly different in their architecture, vascular organization, and cellular composition. Red pulp consists basically of blood cells, including erythrocytes, granulocytes, and circulating mononuclear cells—mainly macrophages. The red pulp macrophages are actively phagocytic and remove old and damaged erythrocytes and blood-borne particulate matter. The white pulp is subdivided into the PALS, the follicles, and the marginal zone. It is composed of lymphocytes, macrophages, dendritic cells, plasma cells, arterioles, and capillaries in a reticular framework similar to that found in the red pulp [21].

We observed that the highest level of iba-1 positive regions in the white pulp of the spleen section compared to the red pulp (Figure 1 and Figure 2A) can be explained by the large number of macrophageal cells of different subpopulations located in the white pulp. At the same time, we detected an increased synthesis of IL-1b and an elevated expression of iNOs in red pulp compared with white pulp after cucumarioside A_2_-2 administration (Figure 2B,C). This observation can be related to the presence in the red pulp of a macrophage subpopulation that is more sensitive to the effects of CA_2_-2. Previously, we showed that CA_2_-2 stimulates only large granulated F4/80+ macrophages with overexpressed levels of purinergic receptors of P2X1 and P2X4 types (4).

In our work, we used the method of laser microdissection for obtaining separate fragments of red and white pulp of the mouse spleen of various experimental groups. We observed a marked shift in the mass spectra of proteins and peptides in different parts of mouse spleen after glycoside and LPS administration in vivo. The characteristic peaks whose intensities significantly changed under the effects of immunomodulation were then examined. Some of these changes occurred in the same manner as for CA_2_-2 or LPS, indicating a similar effect by these compounds. At the same time, a number of changes in protein and peptide profiles were specific for each drug, reflecting the individual characteristics of immunostimulatory effects. It is likely that the observed variations in mass spectrometric peptide/protein profiles, were directly related to the immunostimulatory effects of the test compounds.

There was a significant difference in the mass spectra obtained between the red and white pulp under the action of CA_2_-2. The greatest changes in the peptide/protein profile were found in the red pulp. This may indicate that the glycoside acts more selectively on macrophageal cells, mainly localized in the red pulp and marginal zone of the organ. Further proteomic studies using the separation of splenic proteins by two-dimensional electrophoresis followed by subsequent mass spectrometry detection of regulated protein spots on a gel will allow for the identification of the structure of the molecular targets (biomarkers), the expression of which is regulated by CA_2_-2 in the mouse spleen.

## 4. Materials and Methods

### 4.1. Triterpene Glycoside Isolation

Triterpene glycoside cucumarioside A_2_-2 or 3-*β*-*O*-{3-*O*-methyl-*β*-d-glucopyranosyl-(1→3)-*β*-d-glucopyranosyl-(1→4)-[*β*-d-xylopyranosyl-(1→2)]-*β*-d-quinovopyranosyl-(1→2)-4-*O*-sodium sulfate-*β*-d-xylopyranosyl}-16-ketoholosta-7,25-diene (melting point 245–247 °C. [α]_D_^20^: –68 (*c* 0.1 pyridine)) was isolated from an ethanolic extract of Far-Eastern sea cucumber *C. japonica* using hydrophobic chromatography on polytetrafluoroethylene powder Polychrom-1 (Biolar, Latvia) followed by chromatography on a Si gel column and HPLC as described previously [22]. The purity of the compound was checked by ^13^C NMR and compared with published data. The chemical structure of cucumarioside A_2_-2 and ^13^C NMR spectrum are presented in Figures S1 and S2 of Supplementary Data.

### 4.2. Animals

Female Balb/c mice weighing 20 g were purchased from the RAMS nursery «Stolbovaya» (Russia) and kept at the animal facility under standard conditions. All experiments were conducted in compliance with all rules and international recommendations of the European Convention for the Protection of Vertebrate Animals used for experimental studies. All procedures were approved by the Animal Ethics Committee at the G.B. Elyakov Pacific Institute of Bioorganic Chemistry, Far Eastern Branch of the Russian Academy of Sciences, according to the Laboratory Animal Welfare guidelines. Three groups (6 mice in each group) were used in the experiments: group 1 (control)—animals were treated with PBS; group 2 (experimental)—animals were administrated with CA_2_-2, 3 mg/kg; and group 3 (experimental)—animals were administrated with LPS, 1.5 mg/kg.

### 4.3. Tissue Preparation

A solution of CA_2_-2 or LPS in water was administered *i.p.* once to Balb/c mice. Negative control mice received injections with PBS alone. After 18 h, mice were sacrificed by cervical dislocation, and spleens were then immediately surgically removed within 10 min and fixed for 8 h at 4 °C in 10% phosphate buffered formalin. After rinsing, spleens were processed and embedded in paraffin according to standard embedding techniques and used for further immunohistochemical staining procedures.

### 4.4. Cryosectioning

To prepare tissue for cryostat sectioning, fresh desiccated spleens were gently frozen in liquid nitrogen at −80 °C. Before preparing slices, tissues were equilibrated to −25 °C followed by sectioning at −25 °C. The tissue sample was sectioned at a thickness of 12 μm with Feather C35 80 mm blades (Feather Safety Razor, Osaka, Japan) in a Microm CryoStar NX 70 (Thermo Scientific, Loughborough, UK). Serial longitudinal sections were made and transferred directly onto SuperFrost microscopic glasses for monitoring cell and tissue morphology and on PEN membrane slides for microdissection (Thermo Scientific, Loughborough, UK). The slices were desiccated at RT for 45–60 min on the slide for future use.

### 4.5. Laser-Capture Microdissection

For isolation and harvesting of the different cell types from the tissue sections by laser microdissection, a PALM Laser Microbeam Instrument (Zeiss, Bernried, Germany) was employed. The operational settings for laser focus, laser power, and LPC functions were by the PalmRobo 4.5 software. Cell material was separated from 6 cryosections for each of the three biological replicates (*n* = 3–6/individuals/time point). Cells of red and white pulps were collected into the adhesive lid of a 0.2 μL microtube (Zeiss, Göttingen, Germany) under ×20 magnification. The area of micro-dissected materials for each sample was around 600,000 μm^2^. Collected samples were used immediately or stored at −20 °C until subsequent processing.

### 4.6. Immunohistochemistry

The iba-1, iNOs and IL-1 beta activity of the spleen was assayed and measured using rabbit primary polyclonal antibodies for iba-1 (1:500, ab108539, Abcam, Cambridge, MA, USA), iNOs (1:200, ab15323, Abcam, Cambridge, MA, USA) and IL-1 beta (1:1000, ab9722, Abcam, Cambridge, MA, USA). Secondary antibodies (PI-1000 anti-rabbit (Peroxidase), Vector Laboratories, Burlingame, CA, USA) were used according to the manufacturer’s instructions. PBS was used as a negative control instead of primary antibody in the immunohistochemical reaction. Paraffin sections of spleen (7 μm) after deparaffinization were incubated in 3% hydrogen peroxide for 10 min. After three washes in PBS, sections were incubated for 60 min with 2.5% normal horse blocking serum. Spleen sections were incubated with primary antibodies on glass in a humidified chamber at 4 °C for 24 h. After 3 washes, sections were incubated in a secondary antibody solution for 30 min. After washing, sections were treated for 5–10 min with chromogen (Thermo Scientific, DAB Plus, Waltham, MA, USA). The slices were washed with PBS, dehydrated and embedded in balsam.

### 4.7. Histological Staining

Spleen paraffin-embedded sections (7 μm) were stained with H&E according to the standard procedure for morphological tissue analysis. All preparations were examined by light microscopy (Axio Image Z2, Carl Zeiss, Oberkochen, Germany), images were captured using a digital camera (AxioCam HRc, Carl Zeiss, Oberkochen, Germany) and stored as TIFF files. The resulting images were processed and analyzed using ImageJ software (NIH, Rockville, MD, USA). The area of iba-1-, iNOS-, IL1β-positive macrophages in the white and red pulp spleen immunohistochemical staining was determined using ImageJ software (NIH, Rockville, MD, USA) (plugin: IHC Toolbox). The number of images for counting was at least 60 per group.

### 4.8. Spleen Macrophage Isolation

Mice were sacrificed by cervical dislocation. Isolated spleens were ground with a glass homogenizer in saline solution, and the cell suspension was passed through nylon gas (280 mesh). The resulting suspension of splenocytes was washed from the pellets three times with saline solution by centrifugation (1500 rpm, 5 min, 5 °C) and resuspended in the required amount of saline. Then cell suspension was applied to a Petri dishes and left at 37 °C in an incubator for 2 h to facilitate attachment of macrophages to the dish. Then a cell monolayer was triply flushed with PBS (pH 7.4) for deleting attendant lymphocytes, fibroblasts and erythrocytes and macrophages were obtained using a scraper for further experiments.

### 4.9. ROS Determination

The suspension of macrophages (2–5 × 10^6^ cells/mL) was incubated with 10 μM H_2_DCFH-DA (Invitrogen, Carlsbad, CA, USA) for 30 min at 37 °C in the dark. Following washing with cold PBS, and then analyzed with fluorescent flow cytometer FACScalibur (Becton-Dickinson, Franklin Lakes, NJ, USA). DCF fluorescence was measured at an excitation wavelength of 488 nm and emission at 520 nm. Data acquisition and estimation of results were performed using BD CellQuest Pro (Becton-Dickinson, Franklin Lakes, NJ, USA) and WinMDI 2.9 (TSRI, La Jolla, CA, USA).

### 4.10. NO Measurements

The suspension of spleen macrophages were adhered to glass cover slips for 2 h at 37 °C. Cells were washed 1 time with PBS and repleted with 10 μM DAF-FM diacetate (Alexis Biochemicals, Gruenberg, Germany) for 40 min at 37 °C. Next, cells were washed with PBS, 3 times, and left in the 3rd wash for 30 min at room temperature to allow for de-esterification of the dye. Glass cover slips mounted on glass slides, were viewed by use of a LSM 710 LIVE confocal laser scanning microscope (Carl Zeiss GmbH, Jena, Germany). Fluorescence was excited at 488 nm and emission recorded after 500 nm. The objectives were a Plan-Neofluar 10×/0.3 and LD Plan-Apochromat 63×/1.70 Oil DIC M27. Intensity of fluorescence was quantified using ZEN 2011 software for the Carl Zeiss Laser Scanning Microscope and data was transferred to Microsoft Excel for further calculations. Data were presented as the mean from several separate experiments (at least 900 cells were analyzed in each experiment).

### 4.11. Macrophages Activation and Polarization

RAW 264.7 murine macrophages cultured in DMEM supplemented with 10% FBS, 1% sodium pyruvate and 1% MEM nonessential amino acids at 37 °C under a humidified 5% CO_2_ atmosphere were co-incubated with different concentrations of CA_2_-2 (0.1 and 0.01 μM) and then subsequently by incubating with LPS (1 μg/mL) and IFN-γ (20 ng/mL) for 24 h at 37 °C for observing the change in M1 polarization. A similar procedure was done by replacing IFN-γ with IL-4 (20 ng/mL) for macrophages M2 polarization. Polarized macrophages were collected in microcentrifuge tubes (1 × 10^6^ cells/tube), washed three times with PBS, and then resuspended in 1 mL PBS in FACS tube. Finally, the immunofluorescence of RAW 264.7 cells was identified by anti-CD 86 and anti-CD 206 antibodies (Abcam, Cambridge, MA, USA) with the FACScan flow cytometer (Becton-Dickinson, Franklin Lakes, NJ, USA).

### 4.12. MALDI-TOF-MS

Mass spectra were acquired with an Ultraflex III MALDI TOF/TOF mass spectrometer (Bruker Daltonics, Germany) in the linear positive ion mode with mass range from 2000 to 17,000 *m*/*z*. A pulsed smartbeam laser at a wave length of 355 nm was operated at a frequency of 10 Hz with a delayed extraction time of 150 ns. Each mass spectrum was the average of typically 500 laser shots obtained from several positions within a given sample spot. The sections of the red and white pulp of the spleen cut by means of the laser microdissector were thoroughly mixed with 5 μL of sinapinic acid (SA) matrix and spotted on a stainless-steel MALDI target, then the target was placed into a desiccator for a minimum of 60 min.

Prior to each data acquisition, external calibration was conducted using a protein calibration standard I (Bruker Daltonics, Bremen, Germany). Mixed with matrix, it was deposited separately onto a MALDI target to determine optimum MS parameters and verify the results obtained with tissue samples. SA was dissolved at 10 mg/mL in water/acetonitrile/TFA (49.95/49.95/0.1, vol/vol/vol). The calibration standard samples were directly mixed with the matrix in a 1/1 (vol/vol) ratio. One μL of the mixture was spotted on a stainless steel plate and allowed to dry under ambient conditions. Data were acquired with the Flex Control 3.0 software and processed with the Flex Analysis 3.0 software. The pretreated MS data were used for visualization and for statistical analysis in ClinProTools (demo version 2.0, Bruker Daltonics, Bremen, Germany). Peak statistics have been performed by means of a Welch’s *t*-test.

### 4.13. Statistical Analysis

The data obtained by histological and immunohistochemistry studies were subjected to statistical analysis using one-way ANOVA followed by Tukey’s post hoc test. Data were expressed as mean ± SEM and *p* < 0.05 was considered statistically significant. All statistical tests were performed using the GraphPad Prism 4.00 software.

## Figures and Tables

**Table 1:** Combined analysis of biomarker candidates of two sample classes (white pulp and red pulp) obtained from control and CA_2_-2 treated mice.

White Pulp. Control vs. CA_2_-2				Red Pulp. Control vs. CA_2_-2			
Average Mass	Regulation in CA_2_-2	Fold	*p* Value	Average Mass	Regulation in CA_2_-2	Fold	*p* Value
4618.93	down	1.98	0.00518	10,171.16	up	2.38	0.000221
14,969.48	down	4.75	0.011	15,722.66	up	4.14	0.000923
6109.73	up	15.03	0.0441	15,938.71	up	5.17	0.00154
				15,181.67	up	3.50	0.00157
				16,025.7	up	3.19	0.00261
				15,347.37	up	3.43	0.00261
				15,298.01	up	2.96	0.00308
				15,321.28	up	2.89	0.00578
				14,051.18	up	2.49	0.0166
				14,970.11	up	1.59	0.039
				7874.28	up	1.21	0.039
				7858.9	up	1.22	0.0392
				7483.07	up	1.21	0.0467

**Table 2:** Combined analysis of biomarker candidates of two sample classes (white pulp and red pulp) obtained from control and LPS treated mice.

White Pulp. Control vs. LPS				Red Pulp. Control vs. LPS			
Average Mass	Regulation in LPS	Fold	*p* Value	Average Mass	Regulation in LPS	Fold	*p* Value
5673.7	up	1.29	0.00153	14,974.99	up	3.47	1.1 × 10^−9^
11,315.51	up	1.90	0.00622	15,728.22	up	7.21	1.1 × 10^−9^
7026.31	up	1.50	0.00622	15,186.03	up	7.89	4.88 × 10^−8^
7483.79	down	1.44	0.00622	15,343.1	up	6.55	1.87 × 10^−7^
11,353.64	up	1.92	0.00876	15,937.69	up	10.21	3.1 × 10^−7^
14,055.76	up	1.89	0.00894	16,031.18	up	8.28	1.4 × 10^−6^
5655.6	up	1.31	0.00953	7486.33	up	1.63	1.5 × 10^−6^
13,808.78	up	1.64	0.014	15,301.7	up	5.50	1.5 × 10^−6^
6901.11	up	1.33	0.014	10,173.85	up	2.24	2.48 × 10^−5^
14,019.17	up	1.83	0.0143	14,056.51	up	2.66	0.000209
7007.9	up	1.31	0.0282	14,019.57	up	2.21	0.00158
8454.9	up	1.46	0.0331	7862.17	up	1.27	0.00174
				4966.07	down	1.66	0.00322
				8458.41	up	1.47	0.00322

## Supplementary Materials

The following are available online at www.mdpi.com/1660-3397/15/11/341/s1, Figure S1: The structure and ^13^C NMR spectrum of cucumarioside A_2_-2, Figure S2: The assignment of signals in the ^13^C NMR spectrum of cucumarioside A_2_-2.
