# Supplementary material for: Cucumarioside A2-2 Causes Macrophage Activation in Mouse Spleen
Source: Mar Drugs. 2017 Nov 1;15(11):341. doi: 10.3390/md15110341 (PMC5706031; doi:10.3390/md15110341)
Supplement: Supplementary file 1 [file marinedrugs-15-00341-s001.pdf]

marine drugs

MDPI

SUPPLEMENTARY DATA

Cucumarioside A$_2$-2 Causes Macrophage Activation In Mouse Spleen

Pislyagin E.A.,[1*] Manzhulo I.V.,[2,3] Gorpenchenko T.Y.,[4] Dmitrenok P.S.,[1] Avilov S.A.,[1] Silchenko A.S.,[1] Wang Y-M.,[5] Aminin D. L.[1]

[1] G.B. Elyakov Pacific Institute of Bioorganic Chemistry, Far Eastern Branch of the Russian Academy of Sciences, Vladivostok, Russia
[2] National Scientific Center of Marine Biology, Far Eastern Branch of the Russian Academy of Science, Vladivostok, Russia
[3] School of Biomedicine, Far Eastern Federal University, Vladivostok, Russia
[4] Federal Scientific Center of the East Asia Terrestrial Biodiversity, Far Eastern Branch of the Russian Academy of Sciences, Vladivostok, Russia
[5] Institute of Molecular Medicine and Bioengineering, Department of Biological Science and Technology, National Chiao Tung University, Taiwan
* Correspondence: pislyagin@hotmail.com; Tel.: 7(4232)31-99-32; Fax: 7(4232)31-40-50

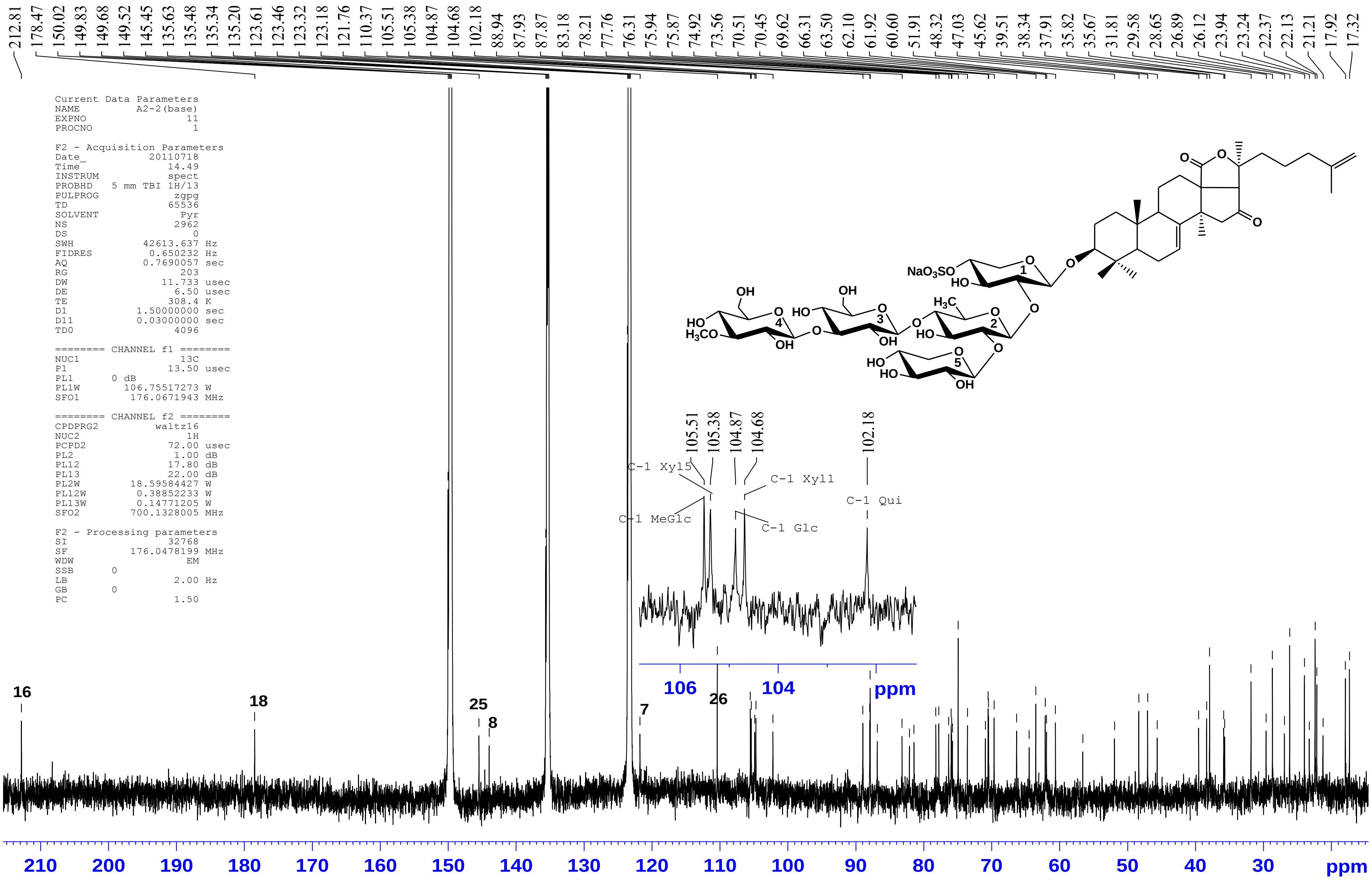

Supplementary Fig. S1. The structure and ^{13}C NMR spectrum of cucumarioside A_2-2.

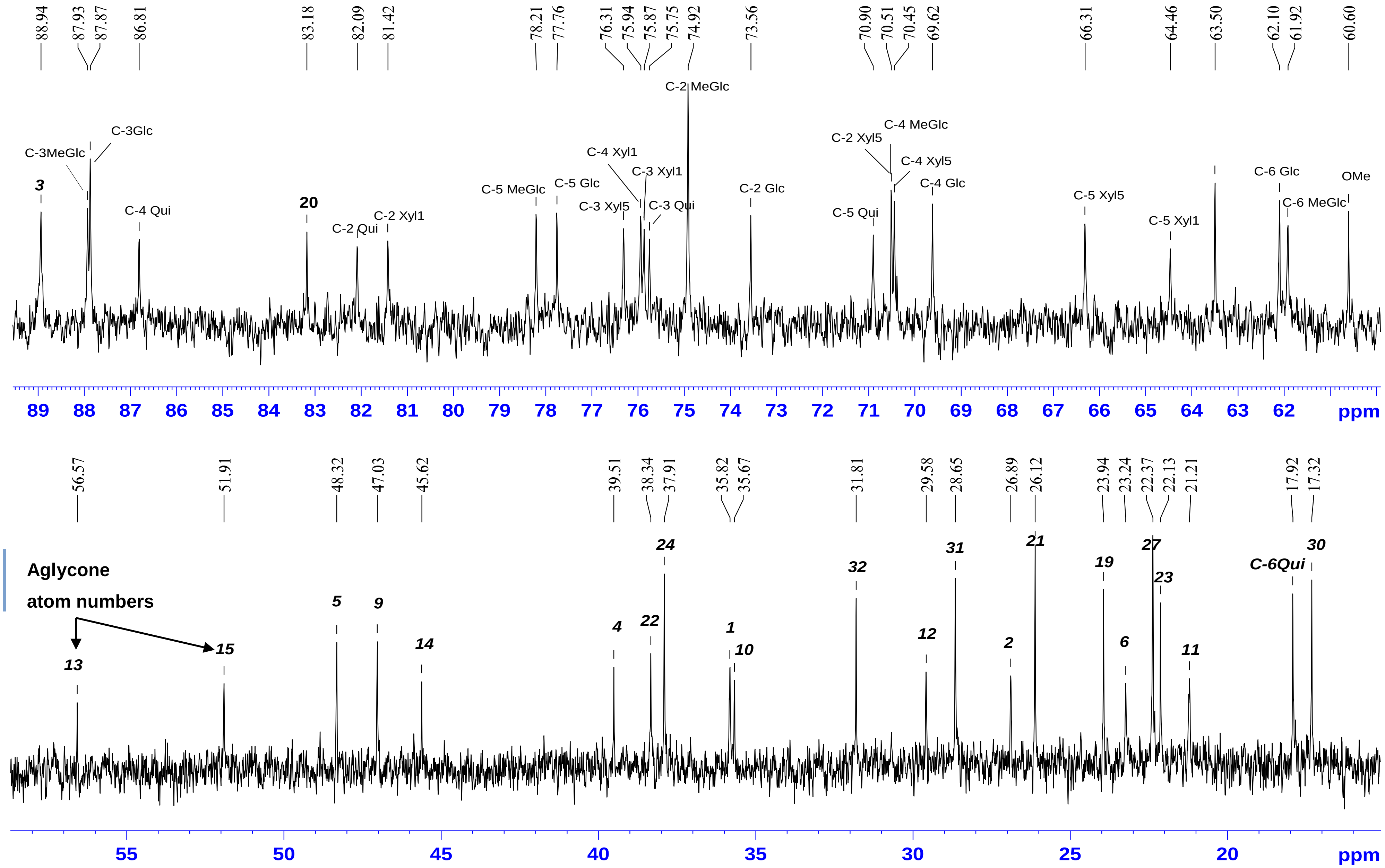

Supplementary Fig. S2. The assignment of signals in the ^{13}C NMR spectrum of cucumarioside A_2-2.
